# Improving instrument detection for a robotic scrub nurse using multi-view voting

**DOI:** 10.1007/s11548-023-03002-0

**Published:** 2023-08-02

**Authors:** Jorge Badilla-Solórzano, Sontje Ihler, Nils-Claudius Gellrich, Simon Spalthoff

**Affiliations:** 1 Institute of Mechatronic Systems, Leibniz University Hannover, Garbsen, Germany; 2 Department of Cranio-Maxillofacial Surgery, Hannover Medical School, Hannover, Germany

**Keywords:** Robot-assisted surgery, Robotic scrub nurse, Surgical instrument detection, Multi-viewpoint inference, Mask R-CNN

## Abstract

**Purpose:**

A basic task of a robotic scrub nurse is surgical instrument detection. Deep learning techniques could potentially address this task; nevertheless, their performance is subject to some degree of error, which could render them unsuitable for real-world applications. In this work, we aim to demonstrate how the combination of a trained instrument detector with an instance-based voting scheme that considers several frames and viewpoints is enough to guarantee a strong improvement in the instrument detection task.

**Methods:**

We exploit the typical setup of a robotic scrub nurse to collect RGB data and point clouds from different viewpoints. Using trained Mask R-CNN models, we obtain predictions from each view. We propose a multi-view voting scheme based on predicted instances that combines the gathered data and predictions to produce a reliable map of the location of the instruments in the scene.

**Results:**

Our approach reduces the number of errors by more than 82% compared with the single-view case. On average, the data from five viewpoints are sufficient to infer the correct instrument arrangement with our best model.

**Conclusion:**

Our approach can drastically improve an instrument detector’s performance. Our method is practical and can be applied during an actual medical procedure without negatively affecting the surgical workflow. Our implementation and data are made available for the scientific community (https://github.com/Jorebs/Multi-view-Voting-Scheme).

## Introduction

In the last decade, a significant scarcity of medical workers has been observed [1, 2], which has been aggravated by the Covid-19 pandemic [3]. This, in combination with the increasing acceptance of healthcare robots [4, 5], motivates the development of robotic scrub nurses (RSNs) as autonomous surgery assistants, which could mitigate staff shortages and become affordable for medical centers.

**Fig. 1:**
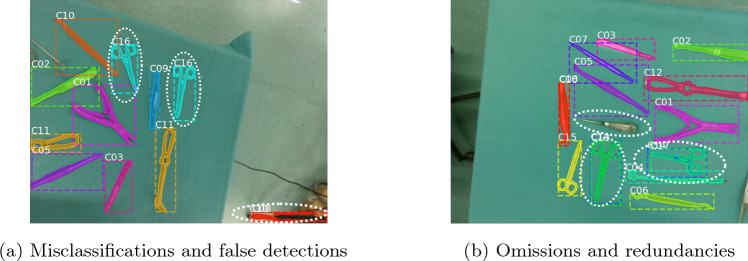
Typical performance errors of instrument detectors based on instance segmentation. **a** Misclassifications occur when an incorrect class is assigned to an instrument, e.g., different tools assigned to the same class (C16). False detections are incorrect predictions associated with the background. **b** Omissions arise when no prediction is made for an instrument. Redundancies occur when multiple predictions correspond to the same tool (double bounding boxes)

Passing the correct requested instrument to the surgeon is a fundamental task of an RSN, thus, instrument detection is of utmost importance. Common strategies for addressing this task rely on deep learning methods [6, 7], and have achieved a high success rate, e.g., 91.2 mean Average Precision (mAP) [8]. Nevertheless, their performance is not perfect. In a high-risk environment such as surgery, the existence of errors can significantly hinder the applicability of an RSN in real-world scenarios. As presented in Fig. 1, typical errors manifest in the form of misclassifications, false detections, omissions, and redundancies.

A way to reduce these errors is to combine data from multiple viewpoints. Several studies have been performed on the topics of multi-view object detection and segmentation. In [9], object detection performance is improved with the integration of images from different viewpoints in X-ray inspection. In [10], multi-view instance segmentation is employed to improve the performance on panoramas, fusing the segmentation results in image space to achieve consistent results. In [11], data from multiple views is employed using a robot manipulator with an attached RGB-D camera to achieve object segmentation and address the challenge of object picking. The findings of these and other studies [12–14] are consistent: the consideration of multiple viewpoints is beneficial for deep-learning-based object recognition.

In this work, we expand upon our previous publication [15] by demonstrating the benefits of using data from multiple views for instrument detection for an RSN, which differs from traditional single-frame instrument detection approaches [6–8]. In contrast with the 3D scene and comparatively larger objects considered in other works [11, 14], we focus on a planar scene, with thin and flat reflective instruments on a table. This makes the use of point-cloud-based segmentation unreliable. Thus, we adopt a simple, yet effective 2D voting scheme to reduce the number of detection errors. The voting is applied using complete predicted object instances, in opposition to the pixel-wise strategies used by other authors [10, 11]. To the best of our knowledge, no other published work for multi-view object detection or segmentation adopts this kind of voting approach.

**Fig. 2:**
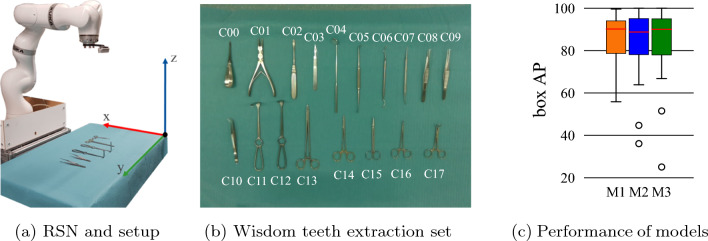
RSN system, surgery set, and models employed during our experiments. **a** The instruments are placed over a surgical cloth on a medical table in front of the robot. An RGB-D camera is used in an eye-in-hand configuration. **b** The instruments of our surgery set are identified by labels from “C00" to “C17". **c** Three Mask R-CNN models (M1, M2, M3) are used during our experiments, with performances of 83.8, 83.3, and 82.6 box mAP, respectively. M1 includes no outliers, while M2 and M3 include two, corresponding to classes with weak performance. Performances are in terms of the box AP, at an IoU of 0.5

Our proposed method exploits the typical setup of an RSN (Fig. 2a), including a robot manipulator and an RGB-D camera in an eye-in-hand configuration. We consider the complete surgery set for wisdom teeth extraction (Fig. 2b), which includes both unique and similar-looking instruments, to create a challenging scenario for our instrument detectors. The viewpoint of the camera can easily be modified by changing the robot’s pose. Image frames and point clouds of the scene can be acquired at each pose. We employ these data, the instances predicted by our detectors, and the robot’s poses to map the locations of the instruments onto a common plane (defined by axes *x* and *y* in Fig. 2a). Overlapping predictions in this plane can be matched together and used as votes. These, in combination with our instance-based voting scheme, can be used to determine the final 2D poses and classes of the instruments on the table plane.

The contribution of this work is the introduction of an instance-based multi-view voting scheme (MVVS) that uses images and point clouds from different views to dramatically enhance the performance of trained instrument detectors. Our strategy is effective even in the presence of outliers with low performance and can be integrated into a real-world operation without negatively affecting the surgical workflow. Our method is practical and simple and does not require modifications in the typical setup of an RSN. We believe our approach constitutes a meaningful step toward eliminating the need for an error-free detector while guaranteeing reliable instrument detection.

## RSN setup for instrument detection

In this section, we describe the main components of our RSN, our instrument set, and relevant information of the instrument detectors used in our experiments.

### Equipment and materials

Our equipment and materials are located in our research laboratory. Our RSN is composed of a robot manipulator and an RGB-D camera in an eye-in-hand configuration (Fig. 2a). More information on our equipment is provided in our previous work [15]. A surgery set for wisdom teeth extraction (Fig. 2b), with 18 different instruments, is selected for our experiments. The instruments are placed on a table on a surgical cloth, depicting the arrangement used during an actual surgery, i.e., random placement, avoiding inter-instrument occlusions.

### Instrument detectors

As introduced in [15], low detection performance for some classes can occur when dealing with similar-looking instruments. To evaluate the robustness of our MVVS against these cases, we use it in combination with three different Mask R-CNN [16] models, namely M1, M2, and M3. These are specifically selected so that they have similar mean performances but differ in the performance of the individual classes, as presented in Fig. 2c. M1 includes no outliers and leads to the best performance. All models are trained exclusively on synthetic data created with the mask-based object insertion (MBOI) method [15]. During the data generation, a collection of 300 background images, as well as collections of 90 single-instrument images per class, are considered. Our balanced training set is composed of 7400 images, including an average of 3100 instances per instrument class, with variations below ± 1% from the average value. The validation set includes a total of 82 annotated real images, with 51 instances per class. For training, a learning rate of 0.001 is used. The description of our hardware and other relevant information about our training is based on the values used in [15].

## Localization and data combination

In this section, we describe the required previous steps for the application of our proposed MVVS. These include the localization of the instruments in the scene and the projection of the predictions onto a common plane.

### Localization

Our RGB-D camera allows for the determination of the point cloud of a given scene. The coordinates of the corresponding points are given in a camera-fixed frame. This, as depicted in Fig. 2a, constitutes a non-inertial reference frame since it moves with the robot’s *end-effector* (EE). In order to successfully locate a point in the scene, the point cloud must be transformed into an inertial frame, i.e., the robot-base frame in our case. Mathematically, a three-dimensional point in the camera-fixed frame ${\varvec{p}}_{\textrm{C}}$, depicted in homogeneous coordinates, can be transformed into a point in the inertial frame ${\varvec{p}}_{\textrm{0}}$ using Eq. (1), as follows:

1 $$\begin{aligned} {\varvec{p}}_{\textrm{0}} = {}^{\textrm{0}}{\varvec{T}}_\textrm{EE} {\varvec{X}}^{-1} {\varvec{p}}_{\textrm{C}} \end{aligned}$$

${}^{\textrm{0}}{\varvec{T}}_\textrm{EE}$ and ${\varvec{X}}$ are homogeneous transformation matrices from EE to the robot-base frame, and from EE to the camera-fixed frame, respectively. ${\varvec{p}}_{\textrm{C}}$ can be directly obtained from the point cloud provided by the camera, while ${}^{\textrm{0}}{\varvec{T}}_\textrm{EE}$ can be determined from the robot’s internal sensors. The calculation of ${\varvec{X}}$ requires the use of a hand-eye calibration procedure [17]. We employ the method proposed by Park et al. [18] to determine ${\varvec{X}}$, allowing for the transformation of the points of the point cloud into the inertial coordinate frame, as given in Eq. (1).

### Projection onto a common plane

**Fig. 3:**
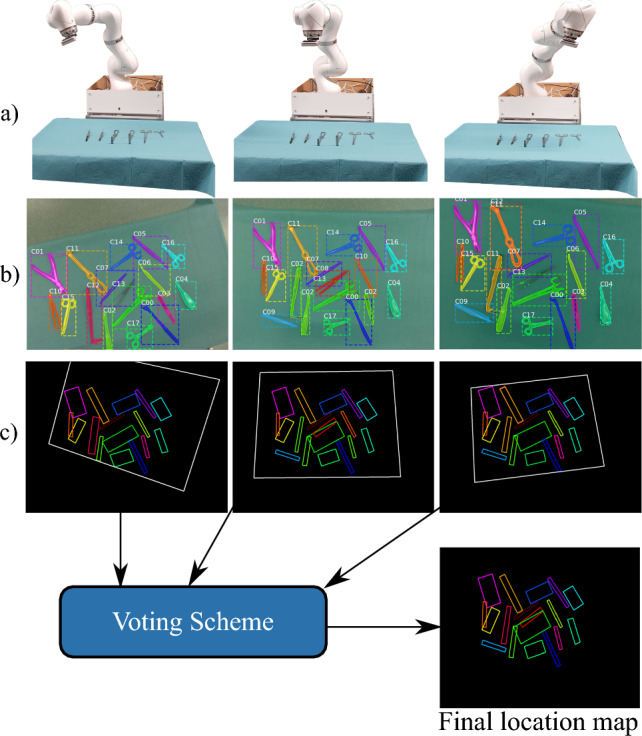
Application of our MVVS for three different viewpoints. **a** RGB images and their corresponding point clouds are recorded from different robot poses. **b** The RGB data is fed to an instrument detector to obtain predictions. **c** The minimal bounding boxes corresponding to the predicted segmentation masks are found and transformed into the robot-base frame to create a location map for each pose. A voting scheme is then used to find a final location map, where the poses and classes of the instruments are described

To determine whether or not predicted instances from different viewpoints correspond to the same instrument, a matching process must be applied. Not only a reliable description of the instruments’ pose (2D position and orientation on the table), but also the projection of the predictions onto a common plane are necessary. We refer to this projection as *location map*. Despite the errors present in the predictions of our models (examples in Fig. 1), it can be noted that the trained instrument detectors successfully predict both the bounding boxes and segmentation masks in most cases. Since the bounding boxes cannot accurately describe the orientation of the instruments, we consider them unreliable to be used as a basis for the location map. Therefore, the projection process is based on the predicted segmentation masks. We perform the mapping by applying the following steps: (1) obtain predictions and point clouds from different robot poses, (2) determine the *minimal bounding boxes* that enclose the predicted segmentation masks, and (3) transform the points of the minimal bounding boxes into the robot-base frame (Eq. 1). Once the projection is complete, a location map is created, in which the projected minimal bounding boxes are referred to as *polygons*. The data corresponding to the instrument class of each polygon is recorded. The information associated with the location maps is fed to our MVVS (Sect. “Multi-view voting scheme”) to determine a final location map that describes the poses and classes of the instruments in the scene. The complete process is illustrated in Fig. 3.

## Multi-view voting scheme

**Fig. 4:**
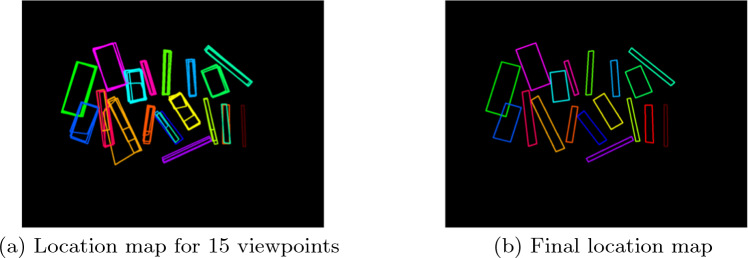
Illustration of the use of multiple viewpoints combined in a single location map (**a**) to create a final location map (**b**)

The generated location maps can be combined into a single map (Fig. 4a). Since the considered data are created with a constant instrument arrangement, the polygons corresponding to each instrument should overlap in this combined location map. We consider a group of overlapping polygons as a *candidate* since they suggest the presence of an instrument in a certain location. Each candidate is associated with a group of *votes*, which are the instrument classes of the overlapping polygons. Our proposed MVVS includes three main steps: (1) matching overlapping polygons to determine the candidates, (2) determining a final polygon to represent each candidate, and (3) estimating the most likely instrument class for the final polygons in the final location map (Fig. 4b). These steps are described in the following subsections.

### Polygon matching

We combine the location maps, as is presented in Fig. 4a. Since our 2D matching problem involves determining the degree of overlap between polygons, our solution is based on the Intersection over Union (IoU) metric, given its capacity to quantify overlapping areas and its standard application in the field of object detection [7, 9, 15]. We define an IoU threshold and match together all polygons with IoU greater than this value. The threshold selection must be investigated since higher values could lead to neglecting matching polygons, while lower thresholds might imply matching together polygons that do not correspond to the same instrument. We explore the optimal threshold values in Sect. “Determination of suitable IoU thresholds”.

### Determination of suitable final polygons

The matched polygons may vary considerably from one location map to the next since some instruments might only appear partially in the field of view of the camera. This can change the size and even the shape of the polygon in the location maps (e.g., in Fig. 4a, blue and light orange polygons on the lower left). Thus, not all matched polygons in a candidate accurately represent the pose of the corresponding instrument. To select representative polygons, we assume that each instrument will be fully visible in most of the frames and that a fully visible instrument will lead to a representative polygon. We perform the selection by finding the polygon that maximizes the sum of its IoU with the other polygons in its corresponding candidate. Mathematically, for a list of polygons corresponding to a candidate ${\textbf{C}}$, the optimal polygon $P_{\textrm{opt}}$ is determined by:

2 $$\begin{aligned} P_{\textrm{opt}} = \underset{P_{\textrm{i}}}{\textrm{arg max}} \left( \sum _{\begin{array}{c} i=0 \\ i \ne j \end{array}}^{n} \textrm{IoU} \left( P_{\textrm{i}}, P_{\textrm{j}}\right) \right) \forall P_{\textrm{i}} \in {\textbf{C}} \end{aligned}$$

### Determination of the final instrument classes

Since our surgery set (Fig. 2b) includes 18 instruments and each instrument corresponds to a class, we match the generated candidates to each of the instrument classes. In our case, the number of candidates is often higher than that of the classes, either due to false detections or incorrect polygon matching. Thus, some of the candidates must often be neglected. We solve this class-assignment problem in the following steps: 1) Create a list of eligible candidates and initialize it with all determined candidates. 2) Find the candidate with the most votes for any particular class and assign it to that class. 3) Remove the assigned candidate from the list of eligible candidates, as well as all votes to the associated instrument class. 4) Repeat steps 2 and 3 until all classes have been matched, and the list of eligible candidates is empty or no votes for the unmatched classes are left. If two or more candidates are considered equally likely to correspond to a class, conflict is determined and no matching is performed for that class.

## Experiments and results

In this section, we describe the process associated with the generation of our data, explain the experiments performed, and analyze the corresponding results.

### Preparation and generation of the data

**Fig. 5:**
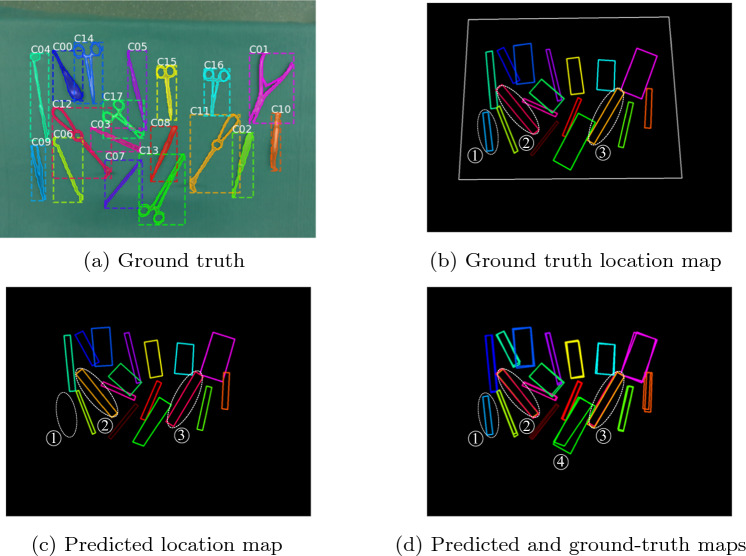
Example of the quantification of the errors during our evaluation. 15 out of 18 instruments are correctly identified. From the ground truth (**a**), a location map is inferred (**b**). When compared with the predicted location map (**c**), errors can be quantified. Three different errors are observed: (1) prediction failure (omission), (2) and (3) are misclassifications. Reasonable difference in matching polygons (4) are not considered errors. The predicted location map is obtained with the application of our proposed MVVS

In order to evaluate our MVVS, we consider 20 different instrument arrangements by placing our 18 instruments in different locations (Sect. “Equipment and materials”). We create collections of data for each of the arrangements, including the RGB images and point clouds corresponding to different viewpoints. The acquisition of these data is performed via a simple motion-capture routine, in which our RSN (Fig. 2a) performs the following steps: (1) The robot moves to an initial pose, i.e., *home pose*, from which all instruments are visible (Fig. 5a). (2) The camera captures an image and the corresponding point cloud of the scene. (3) The robot moves to a new randomly determined pose facing the table, from which a new image and a new point cloud are recorded. (4) The procedure is repeated until the data of 15 different viewpoints are acquired. The changes in the robot’s position coordinates (*X*, *Y*, *Z*) and orientation coordinates (*A*, *B*, *C*) are determined by:

3a $$\begin{aligned} X&= X_{0} + T_{a}u, \hspace{5mm} Y = Y_{0} + 3T_{a}u, \hspace{5mm} Z = Z_{0} + 2T_{a}u \end{aligned}$$

3b $$\begin{aligned} A&= A_{0} + 2O_{a}u, \hspace{5mm} B = B_{0} + O_{a}u, \hspace{5mm} C = C_{0} + O_{a}u \end{aligned}$$

with $u \sim {\mathcal {U}}(-1,\,1)$. Heuristically, we determine that $X_{0} = 500$ mm, $Y_{0} = 0$ mm, $Z_{0} = 650$ mm, $T_{a} = 100$ mm, $A_{0} = -\pi$, $B_{0} = 0$, $C_{0} = \pi$, and $O_{a} = \frac{\pi }{8}$ are suitable values and are therefore used during the data acquisition. The images of each arrangement taken from the home pose are manually annotated and are used as ground truth (Fig. 5a) for evaluation.

### Determination of suitable IoU thresholds

A criterion for the declaration of a *correctly identified instrument* is necessary for evaluation. This implies the existence of matching classes and a minimum degree of overlapping between the predicted and ground truth polygons. An IoU threshold of 0.5 is commonly defined for this matter [8, 14, 15]. In our case, we defined it as 0.3, which is considered reasonable, given our use of minimal bounding boxes and the presence of thin and elongated instruments. An example of error quantification with our MVVS is provided in Fig. 5.

**Fig. 6:**
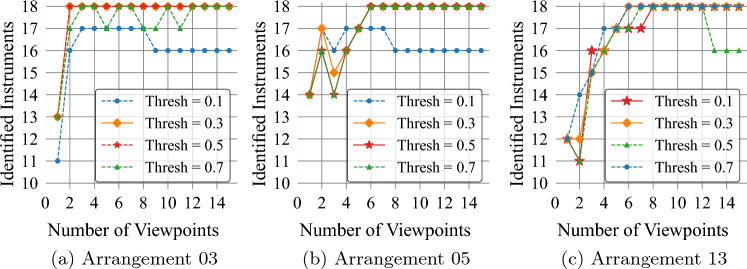
Correctly identified instruments as a function of the number of viewpoints used with our MVVS for model M1. Four IoU thresholds are studied. A general increasing tendency is observed with the use of multiple viewpoints. In these examples, only the threshold values of 0.3 and 0.5 lead to the identification of all 18 instruments using 15 viewpoints or less. A threshold of 0.3 is selected given its faster convergence and superior performance

As described in (Sect. “Polygon matching”), a second IoU threshold is required to match a polygon to a given candidate. As our first experiment, we explore the effect of this threshold on the number of correctly identified instruments for different arrangements (Fig. 2b), using our detector M1. Results for representative arrangements are presented in Fig. 6. The figure indicates how the use of several frames is associated with higher numbers of correctly identified instruments. Intermediate thresholds seem to lead to superior performance since high values tend to neglect polygons, while low values tend to include polygons that might not correspond to the same instrument. An IoU threshold of 0.3 is selected for matching polygons to candidates, given its fast convergence and associated performance. This value is, thus, used in all further experiments.

### Evaluation of the proposed multi-view voting scheme

To evaluate our MVVS, we apply it to the collected data from 20 different instrument arrangements using our instrument detectors, M1, M2, and M3 (Fig. 2c). For our evaluation, we declare our method has achieved *convergence* if the consideration of data from any of the additional viewpoints does not lead to class changes on the final location map. Moreover, a minimum of three additional viewpoints with no associated change is considered, after using all 15 collected pieces of data. Thus, we determine the *minimum number of viewpoints for convergence* (MNVC), which in our cases can range from 1 to 12. With the goal of quantifying the improvement provided by our voting scheme, we determine the number of *errors with a single viewpoint* (ESV), and the number of *errors with multiple viewpoints* (EMV). EMV is defined once convergence is reached. As in our previous experiment, we declare an error when either the overlap between the predicted and ground truth polygons do not meet the minimum threshold of 0.3 IoU or when their instrument classes do not match. In the current experiment, we also quantify the quality of the predictions by calculating the mean IoU (mIoU). The results are shown in Table 1.

The results indicate that the sum of the EMV among all arrangements is drastically lower than that of the ESV for our three models, proving the effectiveness of our method. The reduction in errors experienced by M1 is bigger than that of M2 and M3 (95.9% vs 91.0% and 82.0%, respectively). This difference can be explained by the presence of outliers with lower values (below 50 box AP) for M2 and M3 (Fig. 2c), which indicates a strong tendency for errors in certain classes. This could lead to a relatively lower error reduction after applying our MVVS, explaining the behavior for M1. Moreover, the mIoU of the ground truth and predicted polygons is above 74% in all models, proving the quality of the predicted polygons obtained with the MVVS. M1 achieves convergence with an average of 4.65 MNVC, while M2 and M3 require the use of 6.26 and 7.38 MNVC. We estimate approximately 2.8 s of data gathering and execution time for each viewpoint, implying an investment of less than 15 s for M1.

**Table 1:** Errors for a single viewpoint (ESV) and multiple viewpoints (EMV) using our MVVS. EMV is determined upon reaching convergence

mIoU (%)	Arr	0	1	2	3	4	5	6	7	8	9	10	11	12	13	14	15	16	17	18	19	Sum	Ave
*M1*																							
78.3	ESV	2	4	8	5	6	4	6	3	8	8	3	6	5	6	6	2	2	3	3	5	98	4.90
	EMV	0	1	0	0	2	0	0	1	0	0	0	0	0	0	0	0	0	0	0	0	4	0.20
	MNVC	4	6	5	2	2	5	8	5	3	2	3	4	3	6	5	9	2	9	2	8	93	4.65
*M2*																							
76.4	ESV	8	10	13	5	8	11	8	7	5	1	8	11	5	2	10	0	5	4	7	6	134	6.70
	EMV	0	0	2	0	0	0	0	0	0	2	0	2	0	2	0	1	0	0	0	3	12	0.60
	MNVC	4	5	2	10	12	12	4	5	3	-	7	7	8	12	5	3	3	7	3	7	119	6.26
*M3*																							
74.1	ESV	3	9	6	9	4	8	5	6	8	2	0	11	7	8	9	7	7	2	9	8	128	6.4
	EMV	0	2	1	2	2	2	0	0	1	0	0	1	2	0	1	2	3	1	0	3	23	1.15
	MNVC	2	-	-	3	5	12	4	12	12	2	5	7	-	5	9	-	10	8	4	12	118	7.38

The minimum number of viewpoints for convergence (MNVC) is included, after which no class changes in the final location map occur. The symbol “–” indicates that convergence is not reached with the consideration of 15 viewpoints or less, in which cases EMV is determined with the data of the last considered viewpoint. The instrument arrangements (Arr) are identified by the numbers from “0” to “19”

## Conclusions

With an error reduction greater than 82%, our proposed MVVS proves to be greatly beneficial for the performance of the three considered instrument detectors. Although our method is robust against outliers with low performance, models with relatively high box AP for all classes are recommended (box AP > 50) to optimize its benefits. In combination with our best model (M1), our method identifies correctly 356 instruments out of the 360 included in our image data, for a 98.9 % success rate. Moreover, on average, data from only 5 different viewpoints are required to achieve convergence, implying a time investment of approximately 15 s. This initial investment can be applied at the beginning of surgery to create a reliable map of the instrument arrangement. Since the RSN is meant to hand the instruments to the surgeon, as well as retrieve them, these interactions can be used to modify the created location map after every instrument movement, without the need of gathering additional data. Furthermore, the robot’s idle time can be invested in updating the location map, as verification for additional safety. With this short initial time investment, the exploitation of the idle time, and the modification of the location map according to the instrument movements, our voting scheme should not interrupt the surgical workflow, while guaranteeing high reliability in the instrument detection task. This eliminates the need for an error-free instrument detector.

MVVS could be improved with the consideration of the confusion matrix of the associated instrument detector. The entries can be used to estimate the probability of misclassification, which can be integrated into the determination of the final instrument classes. This will be explored in our future work.
